# Critical Care Ultrasound: Fixer Bestandteil der ICU-Visite?

**DOI:** 10.1007/s44179-022-00027-9

**Published:** 2022-02-11

**Authors:** Gerhard Valicek

**Affiliations:** Klinische Abteilung für Anästhesiologie und Intensivmedizin, Universitätsklinikum St. Pölten – Lilienfeld, Standort St. Pölten, Karl Landsteiner Privatuniversität für Gesundheitswissenschaften, Dunant Platz 1, 3100 St. Pölten, Österreich

Dieser Übersichtsartikel befasst sich mit der routinemäßigen täglichen organübergreifenden Sonografie im Rahmen der Visite auf der Intensivstation, die eine Verlaufsbeurteilung erlaubt.

Der Ultraschall hat durch den technischen Fortschritt und durch die mittlerweile ubiquitäre Verfügbarkeit in allen Fachdisziplinen breite Anwendung gefunden. Neben der formalen umfassenden Anwendung mit kompletter Untersuchung eines Organs, einer Region oder einer physiologischen Einheit haben sich in den letzten Jahren sowohl klinisch symptomorientierte als auch fokussierte organbezogene Untersuchungsalgorithmen durchgesetzt, die eine fächerübergreifende Bedside-Evaluierung von kritisch kranken Patient*innen ermöglichen. So ist zum Beispiel die Akuttherapie eines Schockzustandes heute ohne echokardiographische Evaluierung nicht mehr leitlinienkonform. Ebenso sind die Anlage zentralvenöser Gefäßzugänge und regionalanästhesiologische Techniken nur mehr unter sonographischer Kontrolle empfohlen.

## Qualität in der Medizin

Vor mittlerweile 20 Jahren hat der Konsensusbericht „To err is human“ vom Institute of Medicine in den USA für Aufsehen gesorgt, da in dieser Publikation gezeigt wurde, dass zwischen 50.000 und 100.000 Patient*innen jährlich an den Folgen medizinischer Fehler versterben [1]. Somit reihen sich medizinische Fehler in die Liste der acht häufigsten Todesursachen ein. Die Folge dieser Erkenntnisse war das Projekt „Building a safe health system“ mit dem Ziel der Qualitätsverbesserung in der medizinischen Versorgung. Dazu wurden fünf Bereiche definiert, die die Basis für ein sicheres Arbeiten am Erkrankten bilden [2]:

- Patientensicherheit mit der Entwicklung des medizinischen Risikomanagements
- Effektivität als Maß für den Einsatz evidenzbasierter Methoden
- Effizienz als Hinweis auf die Begrenztheit der Ressourcen
- Rechtzeitigkeit auf die Verfügbarkeit medizinischer Hilfe ohne vermeidbaren Zeitverlust
- Gerechtigkeit, die die Inanspruchnahme medizinischer Hilfe unabhängig von ethnischer Herkunft, Rasse, Vermögensstatus etc. repräsentiert

Im Fokus aller Bereiche sollen die Bedürfnisse des Menschen stehen. Diese gelangen somit in das Zentrum aller Bemühungen.

Qualität in der Intensivmedizin kann über Qualitätsindikatoren definiert werden. Solche wurden von der Deutschen Gesellschaft für Intensivmedizin (DIVI) zuletzt 2017 aktualisiert und publiziert [3]. Sie stellen die Grundlage für eine State-of-the-art-Versorgung dar. Neben einer Reihe anderer medizinischer Indikatoren findet sich hier an erster Stelle die interdisziplinäre multiprofessionelle Visite mit der Definition von Tageszielen (siehe Tab. 1).

**Table 1:** 

Nummer	Hauptindikatoren
I	Tägliche multiprofessionelle und interdisziplinäre klinische Visite mit Dokumentation von Tageszielen
II	Management von Sedierung, Analgesie und Delir
III	Patientenadaptierte Beatmung
IV	Frühzeitige Entwöhnung von einer invasiven Beatmung (Weaning)
V	Überwachung der Maßnahmen zur Infektionsprävention
VI	Maßnahmen zum Infektionsmanagement
VII	Frühe enterale Ernährung
VIII	Dokumentation einer strukturierten Patienten- und Angehörigenkommunikation
IX	Frühmobilisation
X	Leitung der Intensivstation

Quelle: Peer Review Qualitätsindikatoren Intensivmedizin DIVI, 3. Auflage 2017

## Die Visite auf der Intensivstation

Bei der Umsetzung der klinischen Visite im intensivmedizinischen Setting sind verschiedene organisatorische Aspekte zu beachten: Die Informationsweitergabe von einem Dienst zum nächsten kann aufgrund des geltenden Arbeitszeitgesetzes seit 2021 nur mehr im Rahmen komprimierter Übergabevisiten erfolgen, da verlängerte Dienste maximal 25 h dauern dürfen. Selbstverständlich können in diesem Rahmen nur Dynamiken zu Krankheitsverläufen besprochen und nicht sämtliche Details erläutert werden. Somit bekommt die medizinische Wissenskontinuität in der Person einer ärztlichen Stationsleitung eine zentrale Bedeutung. Die Implementierung einer medizinischen Leitung stellt auch den 10. Qualitätsindikator der DIVI dar. Die medizinische Hauptvisite ist somit im Lauf des Vormittags durchzuführen und im Zeitalter von PDMS-Systemen als zweistufige Visite am praktikabelsten. Dabei wird eine „Computervisite“ durchgeführt, bei der sämtliche Informationen aus diversen Subsystemen vereint werden (Radiologiesystem, Laborsystem, Konsile und Befunde aus dem KIS, Mikrobiologie …). In diesem Rahmen werden bereits die nächsten Behandlungsschritte und Tagesziele definiert und dokumentiert sowie die Medikation evaluiert und kontrolliert. Unverzichtbar ist zusätzlich die interprofessionelle Visite am Patientenbett inklusive der klinischen Beurteilung unter Einbindung der Intensivpflege, der Physiotherapie, Logopädie etc. Eine weitere zentrale Aufgabe der Visite ist die Kommunikation mit den Patient*innen. Zusätzlich erfüllt die Visite noch wichtige Aufgaben im Rahmen der Ausbildung unserer Fachassistent*innen. Empfohlen ist die Strukturierung anhand einer Checkliste, die aus folgenden Eckpunkten besteht: Anamnese, Diagnose, aktuelle Vitalparameter und Verlauf, Beurteilung der einzelnen Organsysteme – allgemeine Intensivtherapie – Organisatorisches [4].

Bei den meisten publizierten Checklisten fällt das Fehlen der klinischen Untersuchung auf, die aber einen unverzichtbaren Bestandteil der täglichen Evaluierung darstellt. Genau hier findet die Sonografie Platz für ihre Anwendung und ist gleichzeitig ein Faktor für das Ziel der Qualitätssteigerung in der Intensivmedizin. Das Institute of Healthcare Improvement hat ein Dreiermodell der Qualitätssteigerung entworfen, bei dem die Critical-Care-Sonografie im Zentrum steht. Dadurch können auf Patientenseite Risiken und Strahlenbelastung minimiert, von ökonomischer Seite teure Untersuchungsverfahren und Komplikationsfolgen eingespart werden und auf der Public-Health-Ebene kann die Anwendung Ressourcen schonen, die bis in die Rekonvaleszenz hineinreichen.

## Ultraschall in der Intensivmedizin

Bei sämtlichen Anwendungsgebieten der Sonografie in unserem Fachgebiet findet sich die Abkürzung POCUS für Point of Care Ultraschall. Damit ist gemeint, dass wir bei der sonographischen Diagnostik auf für uns wesentliche Informationen fokussieren, um therapeutische Konsequenzen daraus ableiten zu können. Es geht also nicht um Organsonografie im Sinne der Diagnostik von Raumforderungen oder chronischen Erkrankungen, auch nicht um komplexe Fragestellungen der Echokardiographie, sondern um einfach zu interpretierende bildgebende Informationen, die unser unmittelbares Handeln beeinflussen.

In der Literatur finden sich zahlreiche Untersuchungsprotokolle zur raschen Diagnostik von akuten Problemen. Das RUSH-Protokoll hat die Differentialdiagnose der verschiedenen Schockentitäten zum Ziel, das BLUE-Protokoll zentriert auf das Symptom Atemnot und das FAST-Protokoll dient dem Nachweis freier abdomineller Flüssigkeit bei Trauma-Patient*innen. Bei der routinemäßigen täglichen Evaluierung geht es aber viel mehr um das Dokumentieren von oft relativ kleinen Veränderungen und das Erkennen von Trends. Zu diesem Zweck sind Protokolle hilfreich, die sich an das ABCDE-Schema der Patientenbeurteilung anlehnen und die Gefäßbeurteilung miteinschließen:

- A – Sonografie der Atemwege (mögliches Anwendungsgebiet: Verifizierung der endotrachealen Tubuslage, Guidance bei der dilatativen Tracheotomie)
- B – Lungensonografie (Differentialdiagnostik des interstitiellen Syndroms, Nachweis von Pleuraergüssen und Pneumothorax)
- C – Basisechokardiographie mit Beurteilung des Volumenstatus
- D – Transkranieller Doppler, Beurteilung des Nervus opticus
- E – Abdomineller POCUS (freie Flüssigkeit, Hydronephrose, Gallenblase)

2012 publizierten Manno et al. den Einfluss eines „ICU-sound Protocols“ auf Diagnosestellung und Therapieplan an 125 Patient*innen einer allgemeinen Intensivstation. Die Aufnahmediagnosen wurden in 25,6 % nach Evaluierung adaptiert, in 58,4 % konnte die Verdachtsdiagnose bestätigt werden. Weitere sonographische Evaluierungen veränderten die medikamentöse Therapie in 18,4 % und invasive Prozeduren wurden in 21,6 % indiziert [5].

2015 wurden evidenzbasierte Guidelines zur Anwendung der Critical-Care-Sonografie veröffentlicht, wobei die Autoren in der Einleitung darauf hinweisen, dass die Empfehlungsgrade natürlich nur dem Stand des Evaluierungsdatums entsprechen können und mit zunehmendem Evidenzgrad zu rechnen ist. Die analysierten Daten erlaubten schon zum damaligen Zeitpunkt strenge Empfehlungsgrade für die Diagnostik von Pleuraergüssen, Pneumothoraces, Aszites, Venenthrombosen und zur Anlage zentralvenöser Zugänge [6].

2016 folgten die Guidelines zur Anwendung der Basisechokardiographie in der Intensivmedizin, wobei die höchsten Empfehlungsgrade der Beurteilung des Volumenstatus bei beatmeten Patient*innen und der Detektion einer Perikardtamponade zugeordnet werden konnten. Bei der Beurteilung der links- und rechtsventrikulären Funktion war der Empfehlungsgrad ebenfalls hoch, aber der Evidenzgrad geringer [7].

Ein Expert*innen-Konsensusbericht aus dem Jahr 2011 fasste den Wissensstand über zur Verfügung stehende hämodynamische Monitoringverfahren zusammen und betonte neben den auf dynamischen und klinischen Parametern beruhenden Techniken den Stellenwert der echokardiographischen Evaluierung [8]. Wesentlich ist die Tatsache, dass sich diese Verfahren keineswegs gegenseitig ausschließen bzw. in Konkurrenz zueinander stehen, sondern sich viel mehr ergänzen. Hämodynamische Einschätzung bedeutet also, möglichst viele Puzzlesteine zu sammeln, die zusammengefügt ein korrektes Bild der Kreislaufsituation ergeben sollen, um in Summe das Verhältnis aus Sauerstoffangebot und Sauerstoffverbrauch und den Volumenhaushalt einschätzen zu können.

Der Konsensusbericht definierte auch, welche Eigenschaften Monitoringsysteme erfüllen sollten. Bezüglich der Critical-Care-Echokardiographie gilt es zu betonen, dass nur Basic Skills gefordert sind, die nicht dem Anspruch einer detaillierten kardiologischen Untersuchung gerecht werden müssen. Somit ist die Erlernbarkeit als vorauszusetzende Eigenschaft unter Berücksichtigung standardisierter Ausbildungsprogramme gegeben. Diese bildet die Basis für untersucherunabhängige Interpretation und Reproduzierbarkeit. Die technische Weiterentwicklung erlaubt heute auch bei beatmeten Intensivpatient*innen in den meisten Fällen eine gut interpretierbare Bildqualität in einem der zur Verfügung stehenden Anlotungspunkte. Aufgrund der vielfältigen Anwendungsgebiete der Sonografie (Regionalanästhesie, Guidance bei Interventionen, …) ist die Technik nahezu ubiquitär verfügbar, liefert die Informationen online, ist nebenwirkungsfrei und kosteneffektiv. Die therapeutische Konsequenz durch die Zusatzinformationen hinsichtlich hämodynamischer Einschätzung wurde in zahlreichen Publikationen belegt.

Die COVID-19-Pandemie hat schließlich der Lungensonografie den Stellenwert verschafft, den sie schon seit Jahren innehaben sollte. Bedingt durch die notwendigen Isolationsmaßnahmen mit Minimierung aller Patiententransporte und durch den Vorteil engmaschiger regelmäßiger Reevaluierungsmöglichkeiten ist die sonographische Untersuchung der Lunge und der Pleura zur täglichen Routine in der Intensivmedizin geworden. An unserer Intensivstation, die alle ECMO-pflichtigen COVID-Patient*innen versorgt, hat sich die Anzahl der nativradiologischen Thoraxuntersuchungen um mehr als 50 % reduziert. Um Verläufe und Trends darstellen zu können, ist die Dokumentation von Scores notwendig, zusätzlich besteht an unserer Intensivstation auch die Möglichkeit der Bilddokumentation im Krankenhausinformationssystem.

Sowohl in der internationalen Literatur als auch an unserer Intensivstation hat sich die Dokumentation anhand des LUS-Scores durchgesetzt [9]. Hierbei werden beide Thoraxhälften in jeweils sechs Areale unterteilt und anhand der Untersuchungsergebnisse werden Punktewerte zugeordnet. Dies erlaubt die Schweregrad- und Verlaufsdarstellung für die einzelnen Areale. Der maximale Punktewert mit Konsolidierungen in sämtlichen Arealen beträgt 36, die gesunde Lunge hätte einen Gesamtwert von 0. Der LUS-Score wird an unserer Abteilung standardisiert nach Aufnahme und einmal täglich dokumentiert. Die Ergebnisse fließen in die Adaptierung der Beatmung, der Flüssigkeitstherapie und in die Indikation der kinetischen Therapie ein. Durch den unterschiedlichen Charakter der Aerobronchogramme bei Viruspneumonie und bakterieller Pneumonie kann die Lungensonografie auch als zusätzlicher Puzzlestein zum Nachweis einer Superinfektion dienen [10].

2020 publizierten Hussein et al. ein internationales Expert*innen-Konsensuspapier nach Datenanalyse von 214 Studien für den Einsatz von POCUS bei COVID-19-Patient*innen. Unterschieden wird in dieser Publikation zwischen reiner Lungensonografie und Multi-Organ-POCUS, wobei die Empfehlungen für moderate bis schwere Krankheitsverläufe einer COVID-19-Infektion gelten [11]. Zu folgenden Anwendungsgebieten wurde Stellung genommen und Empfehlungsgrade ausgesprochen:

- Indikation zur stationären Aufnahme
  - POCUS wird zur Triage und Risikostratifizierung empfohlen: LQE (Quality Level of Evidence) II‑B, LA (Level of Agreement): gut
- Diagnosestellung der Pneumonie (Phänotyp)
  - In Kombination mit klinischer Einschätzung: LQE: II‑B, LA: sehr gut
- Kardiovaskuläre Einschätzung
  - Fokussierte Echokardiographie wird sowohl zur initialen Einschätzung, zur Akutdiagnostik akuter hämodynamischer Instabilität, zur Beurteilung der rechts- und linksventrikulären Funktion und zum weiteren hämodynamischen Management empfohlen: LQE: II‑B, LA: sehr gut
  - Die gemeinsame Interpretation von POCUS-Befunden und hämodynamischen Monitoring-Verfahren wird empfohlen: LQE: II‑B, LA: sehr gut
- Screening nach thromboembolischen Ereignissen
  - Aufgrund der immunothrombotischen Dysregulation im Rahmen einer COVID-19-Erkrankung wird ein regelmäßiges Screening nach tiefen Beinvenenthrombosen empfohlen, ebenso die Suche nach Zeichen eines akuten Cor pulmonale bei hämodynamischer Instabilität: LQE: II‑B, LA: sehr gut
- Adaptierung des respiratorischen Supports inkl. Diagnose potenzieller Komplikationen
  - In Kombination mit respiratorischem Standardmonitoring wird der Lungenultraschall höherwertiger als das Thoraxröntgen und gleichwertig zum CT empfohlen: LQE: II‑B, LA: gut
  - Regelmäßige Evaluierungen mit Lungenultraschall bei COVID-19-Patient*innen sind empfohlen: LQE: II‑B, LA: sehr gut
  - Bei respiratorischer Verschlechterung und zur Therapiekontrolle wird POCUS über der alleinigen Lungensonografie empfohlen: LQE: II‑B, LA: sehr gut
  - Zur Diagnostik eines Pneumothorax und einer Ventilator-assoziierten Pneumonie wird die Lungensonografie empfohlen: LQE: II‑B, LA: sehr gut
  - Bezüglich Weaning und Weaningversagen wird POCUS zur Hilfestellung empfohlen: LQE: II‑B, LA: sehr gut
- Einschätzung des Flüssigkeitshaushalts
  - Diagnose der schweren Hypovolämie: LQE: II‑B, LA: sehr gut
  - Lungenultraschall als alleiniges Instrument zur Diagnose kardialer Dekompensation bei COVID-19-Patient*innen wird nicht empfohlen, ebenso nicht als alleiniges Instrument zur Steuerung der Volumentherapie: LQE: II‑B, LA: sehr gut
- Verlaufsbeurteilung und sekundäre Organdysfunktion
  - Identifikation prä- und postrenaler Ursachen des akuten Nierenversagens mittels POCUS wird empfohlen: LQE: II‑B, LA: sehr gut
- Infektionskontrolle/Technologie/Protokolle
  - Tablet-basierte Technologien werden aufgrund der einfacheren Wischdesinfektionsmethoden empfohlen, ebenso wie standardisierte Untersuchungsprotokolle und telemedizinische Qualitätskontrollen: LQE: II‑B, LA: gut

Auf neurointensivmedizinisches Monitoring fokussierte Methoden wird in dieser Arbeit nicht eingegangen, auch die Zwerchfellsonografie als Weaning-Prädiktor ist nicht Gegenstand dieses Expertenkonsens.

Die verschiedenen Anwendungsmöglichkeiten der Sonografie in der Intensivmedizin finden somit – verstärkt durch die Pandemiesituation – breiten Raum in der Literatur und die Empfehlungen bilden eine ausreichende Basis für die Umsetzung in der Praxis. Die tägliche Sonografie erlaubt die Detektion geringfügiger Befunddynamiken und die Darstellung von Trends in Beziehung zu therapeutischen Maßnahmen. Für die routinemäßige Einbindung in die tägliche Praxis scheint ein Kompromiss aus vertretbarem bzw. bewältigbarem Zeitaufwand und einem standardisierten Untersuchungsgang, der die wesentlichen Informationen garantiert, relevant.

**Figure 1:**
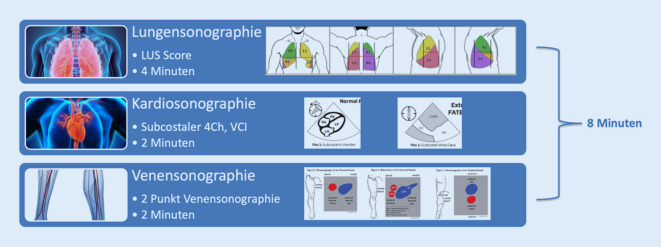

Als Basisuntersuchung im Rahmen der täglichen Evaluierung stellt sich der Untersuchungsgang aus Abb. 1 als praktikabel heraus. Acht Minuten Untersuchungszeit erscheinen bewältigbar, allerdings summieren sich diese Zeiten auf einer 10-Betten-ICU auf 80 min. Daraus resultiert ein nicht unbeträchtliches Ausmaß an Personalbindung und Inanspruchnahme einer Zeitressource, die ärztliche und pflegerische Tätigkeit am Erkrankten beeinflussen. Dieser Untersuchungsgang hat keinerlei Anspruch auf Vollständigkeit. Zusätzliche sonographische Diagnostik (Neurosonografie, Zwerchfellsonografie, Abdomensonografie …) sind speziellen Fragestellungen vorbehalten. Aus meiner Sicht stellt die zeitökonomische Einbindung in den Tagesablauf einer Intensivstation eine große Herausforderung dar.

Benneth et al. publizierten 2008 eine Pilotstudie, um den Workload zweier verschiedener Einbindungsmodalitäten von POCUS zu untersuchen. Einerseits einen sequenziellen Untersuchungsgang, bei dem die physikalische Untersuchung der Patient*innen primär und im Anschluss die sonographische Untersuchung folgte, und andererseits ein paralleler Untersuchungsgang, bei dem im Rahmen der physikalischen Aufnahmeuntersuchung auch der Ultraschall zum Einsatz kam. Grundgerüst für beide Varianten war die Patientenevaluierung nach dem ABCDE-Schema. Zehn Anlotungspunkte waren obligatorisch, sechs weitere speziellen Fragestellungen vorbehalten. Die Studienteilnehmer*innen waren in Ausbildung an der Mayo-Klinik und hatten alle eine zertifizierte POCUS-Ausbildung absolviert. Der Workload wurde anhand des NASA-TLX-Fragebogens evaluiert, der physische, mentale, zeitliche Anforderungen und Erfolg, Anstrengung und Frustration erhebt. Der Workload für den sequenziellen Untersuchungsgang wurde signifikant geringer angegeben als für den parallelen Untersuchungsgang, obwohl der zeitliche Aufwand nicht differierte. Die mentale Anforderung und der Frustrationslevel waren hier ausschlaggebend [12]. Die Ergebnisse erlauben keinen direkten Schluss auf routinemäßige Ultraschalluntersuchungen im Rahmen der ICU-Visite. Trotzdem sind Aspekte der Praktikabilität und Akzeptanz wesentliche Faktoren in der Implementierung und dauerhaften Umsetzung neuer Untersuchungsmodalitäten.

## Implementierung in den Tagesablauf

Die Datenlage zur Implementierung einer sonographischen Evaluierung in die tägliche Intensivvisite ist beschränkt.

Eine internationale Multicenter-Umfrage beschäftigte sich mit dem Thema der Einbindung fokussierter Sonografie in den Tagesablauf der Intensivstationen. Dabei gaben 43 % der Teilnehmer*innen an, dass POCUS nach der Visite durchgeführt wird, 45 % implementieren die sonographische Diagnostik direkt in den Ablauf der multiprofessionellen Visite. Die restlichen 12 % handhaben die Einbindung variabel [13].

Aus meiner Sicht bietet die Diagnostik während der Visite folgende Vorteile:

- Das systematische Durchbesprechen eines Krankheitsverlaufs nach der Visitencheckliste fokussiert auf die aktuell relevante Problematik und bringt die sonographischen Befunde in den momentanen Kontext.
- Weiters führt die unmittelbare Interpretation zu indizierten Therapieänderungen und potenziell zur Planung weiterer diagnostischer Verfahren.
- Diese Informationen stehen automatisch allen Mitgliedern des Behandlungsteams zur Verfügung, die Untersuchung erfolgt qualitätskontrolliert unter Anwesenheit der medizinischen Stationsleitung und ist Teil einer multiprofessionellen Ausbildung.
- Durch die Anwesenheit des Pflegepersonals können notwendige Lagerungsmaßnahmen durchgeführt werden, um die Untersuchungsbedingungen zu optimieren.

Dem gegenüber steht die längere Visitendauer und die dadurch möglicherweise beeinträchtigte Teamakzeptanz. Untersuchungen außerhalb der Visite unterliegen weniger dem Zeitdruck, beeinflussen aber auch andere Prozesse wie zum Beispiel die Pflegetätigkeit. Als größten Nachteil empfinde ich die Trennung von Diagnose und unmittelbarer Therapieentscheidung.

Eine prospektive Beobachtungsstudie in China vergleicht die routinemäßige Ultraschalleinbindung während der Visite mit der rein spontan indizierten Anwendung von POCUS an einer Intensivstation in einem Zeitraum von zwei Jahren. Eingeschlossen waren septische Patient*innen, die definierten Endpunkte waren Outcome-Parameter. In der Analyse zeigten sich die Beatmungsdauer und die Intensivaufenthaltsdauer in der Interventionsgruppe statistisch signifikant kürzer. Als mögliche kausale Erklärung wurde eine restriktivere Flüssigkeitstherapie identifiziert [14].

## Zusammenfassung

Die Anwendung von Point of Care Ultraschall (POCUS) in Form von Critical Care Ultrasound weist einen hohen Empfehlungsgrad auf und verbessert die Versorgungsqualität in der Intensivmedizin. Wenn man die physikalische Patient*innen-Untersuchung als unverzichtbaren Bestandteil der Visite betrachtet, ist die Sonografie als „fifth pillar“ zusätzlich zu Auskultation, Inspektion, Perkussion und Palpation fixer Bestandteil dieses Procederes [15]. In Zukunft ist mit einer Erweiterung der Anwendungsgebiete (Zwerchfellsonografie etc.) zu rechnen, womit der Einsatz von Ultraschall per se für mich einen eigenen Qualitätsindikator der Intensivmedizin darstellt. Die Art der Implementierung in den Tagesablauf muss nach dem Prinzip „One doesn’t fit for all“ individuell an die Prozesse der einzelnen Intensivstationen angepasst werden.
